# Optimizing chillies *(Capsicum annum L*.) germination rate and early seedling performance through nutrient seed priming with potassium nitrate and zinc oxide

**DOI:** 10.3389/fpls.2025.1535305

**Published:** 2025-05-30

**Authors:** Mufunwa Maphalaphathwa, Adornis Dakarai Nciizah

**Affiliations:** ^1^ Agricultural Research Council - Natural Resources and Engineering, Arcadia, Pretoria, South Africa; ^2^ Department of Agriculture and Animal Health, University of South Africa, Florida Park, Roodepoort, South Africa

**Keywords:** Nutrient concentration, priming duration, fresh seedling biomass, crop development, germination rate

## Abstract

Chillies are an economically important crop in South Africa, with increasing demand for both domestic consumption and export. However, smallholder production remains low due to environmental and economic constraints, particularly poor seed germination and weak seedling vigor, which limit fruit quality and yield. Agrotechnological developments, such as seed priming, which is a crucial pre-sowing treatment, can enhance crop establishment and ensure yield consistency. Seed priming, particularly nutrient seed priming (NSP), enhances germination and seedling establishment in various crop species, including *Capsicum annum L*. Although nutrient seed priming (NSP) has improved germination in various crops, species-specific optimization of priming duration and concentration is essential. Currently, no standardized priming protocols exist for chillies in South Africa, necessitating further research, An 8 x 3 factorial experiment was conducted under laboratory and glasshouse conditions to evaluate the effects of eight priming treatments (20 mg/L ZnO, 10 mg/L ZnO, 15 mg/L ZnO, 5 mg/L ZnO, 10 g/L KNO_3_, 5 g/L KNO_3_, 2.5 g/L KNO_3_, and H_2_O) at three priming durations (6, 12, and 24 h) on chilli seed germination and seedling growth. ZnO and KNO_3_ significantly (p< 0.05) influenced germination and early growth parameters. Under laboratory conditions, priming with 20 mg/L ZnO resulted in the highest germination rates, with optimal priming durations of 12 h for ZnO and 24 h for KNO_3_. In the glasshouse, priming with 20 mg/L ZnO for 6 h improved seedling vigor, including shoot height, root length, and biomass accumulation. These findings indicate that ZnO priming, particularly at 20 mg/L for 6 h, is a promising strategy to enhance chilli seedling establishment under South African conditions, potentially improving productivity for smallholder farmers. The results showed that the optimal combination of NSP varies with specific germination indices. Under laboratory conditions, priming with 20 mg/L ZnO led to higher germination rates than KNO_3_ with optimal priming durations of 24 hours for KNO_3_ and 12 hours for ZnO. In the glasshouse, priming seeds with 20 mg/L ZnO for 6 hours produced the best results across most indices demonstrating that priming with ZnO in particular, could be an effective strategy for enhancing early growth and vigor in chillies seedlings.

## 1 Introduction

Chillies (*Capsicum annum L*.) are an economically important crop in South Africa, with high market demand. However, smallholder farmers face significant challenges in achieving optimal production due to economic constraints and environmental factors (Kumar et al., 2021). Optimal chilli production relies on rapid and uniform seed germination, yet this process is often hindered by low germination rates and poor seedling vigor. Factors such as seed coat thickness, capsaicin content, and environmental conditions contribute to irregular germination and weak seedling establishment, ultimately affecting fruit yield and quality (Mpanza and Mavengahama, 2018; Saini et al., 2021; Tu et al., 2022). Seedling emergence and early growth are critical for crop establishment, as they enhance resource acquisition, competition, and overall plant vigor (Seiwa, 2000; E’rahim et al., 2021). Rapid and uniform emergence improves root and shoot development, promotes weed suppression, and ensures consistent fruit maturity, all of which are essential for smallholder farming success (Bianchi et al., 2019; McLachlan et al., 2023).

Therefore, there is a need for low-cost solutions to address these limitations and promote successful chilli production. Significant improvements in seed germination have been reported across many crops with seed priming e.g. maize (Nciizah et al., 2020) and sorghum (Murungu, 2011). Despite these positive findings in other crops, the use of seed priming chilli seeds has not been explored in South Africa especially in the context of small-scale farmers. Seed priming is a pre-sowing treatment that enhances seed performance by improving germination speed, seedling vigor, and stress tolerance (Khaba et al., 2020). Nutrient seed priming (NSP), which involves soaking seeds in nutrient solutions such as potassium nitrate (KNO_3_) or zinc oxide (ZnO), has been shown to enhance germination and early growth in various crops (Mukherjee et al., 2022). However, the effectiveness of NSP depends on crop-specific factors, including priming duration and nutrient concentration, which remain un-optimized for chilli seeds in South Africa

Several seed priming treatments have been developed and tested on various crops over the years. For instance, significant success has been achieved with nutrient seed priming, a method that involves soaking seeds in nutrient solutions to improve their germination and early growth. It is essentially the application of micronutrients to plant seeds by soaking them in a solution of the nutrient of interest for a given duration (Mukherjee et al., 2022). It has been shown to improve germination percentage, germination rate, and days to germination (Nciizah et al., 2020). Additionally, seed priming has been found to reduce anti-nutrient compounds, such as phytic acid, through the soaking process (Poudyal et al., 2023). However, the duration and concentration levels of seed priming are critical factors that determine the success of the process (Nciizah et al., 2020). Nutrient seed priming is therefore a promising agricultural technique that has the potential to enhance seed germination, seedling growth, and overall plant performance. By optimizing the duration and concentration levels of nutrient solutions used in seed priming, researchers and farmers can harness the full potential of this method to improve crop yields, nutrient uptake, and stress tolerance in plants.).

Seed priming with potassium nitrate (KNO_3_) and zinc oxide (ZnO) has been shown to affect the germination and early growth of chilles, with specific characteristics such as percentage, seedling height, and number of leaves indicating the chillies ability to thrive under field conditions (Anosheh et al., 2011; Ruttanaruangboworn et al., 2017; Gordana et al., 2024). Previous research has demonstrated the positive impact of seed priming on other crop species, such as *Zea mays*, where priming with *Plantago ovata* extract improved growth-related attributes, photosynthetic apparatus, and nutrient uptake (Anosheh et al., 2011). However, the effectiveness of priming with KNO_3_ and ZnO depends on various factors such cultivar, duration of soaking and concentration of nutrients. Despite the demonstrated benefits of seed priming in crops such as maize and sorghum (Nciizah et al., 2020; Murungu, 2011), its application in chilli production has not been well explored, particularly in South African smallholder systems. The optimization of KNO_3_ and ZnO priming conditions for chilli seeds remains unclear. This study aims to determine the optimal duration and concentration of these priming treatments to maximize chilli seed germination, seedling vigor, and early growth performance.

## 2 Materials and methods

Laboratory and glasshouse experiments were conducted in 2023 at the Agricultural Research Council– in Pretoria, Gauteng, to determine optimal concentrations and priming durations of ZnO and KNO_3_. The laboratory experiments were conducted at the Agricultural Research Council–Natural Resources & Engineering (ARC-NRE) campus in Arcadia Pretoria, whilst the glasshouse study was conducted at the Agricultural Research Council–Vegetable, Industrial and Medicinal Plants (ARC-VIMP) in Roodeplaat, Pretoria. Minimum/maximum ambient temperatures were kept constant at 13/25°C, with maximum temperatures controlled using thermostatically activated fans in the glasshouse.

### 2.1 Plant material sources

Chilli seeds (Hot Chilli Mix) used in this study were obtained from Starke Ayres (Pty). This variety, Pepper Hot-STAR 6603 F1 hybrid seed, is characterized by a germination period of 16–18 days and reaches maturity in 75–80 days.

### 2.2 Soil description and soil sampling

The soil used for the glasshouse trial was obtained from the ARC-VIMP in Roodeplaat. Soil samples were collected from 0 to 20 cm depth after removing surface litter, using a clean stainless-steel spade to avoid contamination. The samples were thoroughly homogenized to create a composite sample, air dried and sieved through a 2 mm sieve. Subsamples were then transported to the laboratory in clear polythene sampling bags for analysis. The following physio-chemical properties were analyzed; soil pH, texture, exchangeable bases (Ca, Mg, K and Na), soil organic carbon (SOC), electrical conductivity (EC), and cation exchangeable capacity (CEC), Phosphorus (P). Soil pH was measured in water in a 1:2.5 soil water ratio using a pH meter (model pH 25, Crison Instruments) after shaking the suspension for 30 minutes and equilibrated for 30 minutes (Okalebo et al., 2000). EC was determined from the same suspension used for pH. Particle size distribution was measured using the pipette method after oxidizing SOM with hydrogen peroxide (Gee and Or, 2002). Soil organic carbon was determined using the Walkley-Black chromic acid wet oxidation method (Nelson and Sommers, 1996). Exchangeable bases (Ca, Mg, Na, and K) were determined by treating samples with 1M ammonium acetate buffered at pH 7.0 while CEC was calculated from the cations. Phosphorus was determined following Bray 1 method (Nelson and Sommers, 1996; Okalebo et al., 2000).

### 2.3 Laboratory experiment

Seeds were primed with various potassium nitrate (KNO_3_) and zinc oxide (ZnO) solutions at varying concentrations of the nutrient solutions for different durations (6h, 12h, and 24h). These nutrients were selected based on their known ability to enhance germination in other crops (Khafagy et al., 2017; Neto et al., 2020; Sharma et al., 2021). A completely randomized design (CRD) arranged in an 8 × 3 factorial treatment structure was used for the laboratory experiment. Eight nutrient seed priming treatments [5 mg/L ZnO, 10 mg/L ZnO, 15 mg/L ZnO, 20 mg/L ZnO, 10 g/L KNO_3_, 5 g/L KNO_3_, 2.5 g/L KNO_3_, H_2_O (control)] and three priming durations (6h, 12h and 24h) were tested. Each treatment was replicated three times. These concentrations were selected based on previous studies demonstrating their potential to improve germination on other plant species (Neto et al., 2020; Choukri et al., 2022). Similar concentrations were also used by Rai-Kalal and Jajoo (2021) and Ruttanaruangboworn et al (2017) Seeds for the control treatment (0%) were soaked in distilled water (hydro-priming) for the same duration.

ZnO priming solutions were prepared by dissolving the appropriate amount of ZnO powder in distilled water to achieve the desired concentrations, 5mg/L, 10 mg/L, 15 mg/L and 20 mg/L. The calculated mass of ZnO was added in distilled water and stirred with a magnetic stirrer thoroughly to ensure complete dissolution. KNO_3_ solutions were also prepared by dissolving the required mass of KNO_3_ in distilled water to achieve the desired concentrations 2.5 g/L, 5 g/L and 10 g/L.

Thirty chilli seeds were soaked in 100 ml ZnO and KNO_3_ solutions or distilled water (hydro-primed) for 6h, 12h and 24h. Afterwards, the seeds were rinsed three times with deionized to remove the leachates from the seed coat. The seeds were then dried at room temperature for 1 hour as described by Harris et al. (2008), Khafagy et al., 2017; Ullah et al., 2020 and Sharma et al., 2021. Thereafter, 30 seeds per replicate were placed between layers of germination paper in 24 petri dishes and incubated at 25°C for 20 days (Corbineau et al., 2023). The petri dishes were irrigated with 10 ml of distilled water every other day to maintain moisture without overhydration. Seed germination was recorded daily with seeds being considered to have germinated when at least 2 mm long radicle protruded through the seed coat. Days to germination were recorded when 50% of the seeds had germinated (Ullah et al., 2020).

### 2.4 Data collection

Data was collected on the following parameters using equations described by Ellis et al. (1981), Scott et al. (1984), ISTA (1996), and Zahedifar and Zohrabi, (2016) (Equations 1–5). Where *n_i_* is the number of seeds emerged on an *i*th day and d*i* is the number of days counted from the beginning of the experiment. j is set to 14 days in this experiment, n is the number of seeds germinated on day and d is the number of days from the beginning of the experiment, G_1_ – G_n_ is the number of germinated seeds from the first to the last day.

i. Final germination percentage (GP) as follows:


(1) $$GP=\left(\frac{Number\;of\;normal\;germinated\;seeds}{total\;number\;of\;seed}\right)\;\times 100$$

ii. Germination rate (GR) as follows:


(2) $$GR=\sum_{i-1}^{j}\frac{n_{i}}{d_{i}}$$

iii. Mean germination time (MGT) as follows:


(3) $$MGT=\;\frac{\sum^{}n.d}{\sum^{}n}$$

iv. Coefficient of velocity of germination (CVG) as follows:


(4) $$CVG=\;\frac{G_{1\;}+G_{2\;\;}+G_{3}\ldots \ldots .\ldots +\;G_{4}\;}{\left(1\;\times \;G_{1}\right)+\;\left(2+\;G_{2}\right)+\;\left(n+\;G_{n}\right)}$$

v. Emergence percentage (EP) was calculated when cumulative emerged seeds with normal radicle and plumule are visible using the following equation:


(5) $$EP=\;\left(\frac{Number\;of\;normal\;emerged\;seeds}{total\;number\;of\;seed}\right)\;\times 100$$

vi. Days to emergence (DE), chlorophyll content index (CCI), stem diameter (SD), seedling height (SH), Fresh seedling weight (FSW) and dry seedling weight (DSW) and final root length (RL) were determined. Stem diameter was measured with a digital Vernier calliper, chlorophyll content with a chlorophyll meter (MINOLTA SPAD-502) and seedling height was measured using a ruler. On the final day of the experiment, seedlings were uprooted and washed off over a 53 μm sieve to remove all the soil from the roots. Root length was measured with a ruler whilst FWS was measured with a weighing balance. Dry seedling weight mass was measured after drying the seedlings at 65 °C in a forced air oven until constant weight was achieved.

### 2.5 Glasshouse experiment

Results from the laboratory were used to formulate greenhouse treatments. The best performing concentrations from the laboratory experiment were selected: ZnO at 10 mg/L and 20 mg/L and KNO_3_ at 10 g/L. Additionally, positive control (hydro-priming) and negative control (non-priming) were included. The three priming durations (6, 12 and 24h) from the laboratory study were maintained. A completely randomized design (CRD), arranged in a 5 x 3 factorial treatment structure was used for the glasshouse experiment. The first factor was seed priming at five levels (ZnO at 10 mg/L, and 20 mg/L, KNO_3_ at 10 g/L, hydro-priming (positive control), non-priming (negative control), while priming durations (6, 12 and 24h) made up the second factor. Each treatment was replicated three times resulting in a total of 15 treatment combinations (5 priming treatments x 3 durations).

Experimental pots were prepared by filling each pot with soil sieved through a 2 mm sieve. NPK fertilizer was added to all pots, including the control pots at the recommended rate for chilli cultivation. Seed priming was done following the same procedures as those used for the laboratory. Three seeds of each treatment were sown at depths of 0.03 m in 25 cm diameter plastic pots. Pots were irrigated with 500 ml tap water every two days. The experiment was conducted in a climate-controlled glasshouse, where the air temperature was maintained at 25 ± 3°C using an automated heating and ventilation system. Relative humidity was kept between 60% and 80% through regular misting, while light intensity was supplemented with artificial LED grow lights to ensure optimal photosynthetic activity. The experiment was run for a period of six weeks before termination.

### 2.6 Statistical analysis

The effects of nutrient seeds priming concentration and soaking duration on chillies germination indices were analyzed using analysis of variance (ANOVA) (Gomez and Gomez 1984) using JMP version 17 pro statistical software (SAS Institute, Inc., Cary, NC, USA, 2022). A 2-way ANOVA was used to analyze data for both the laboratory experiment, and the glasshouse experiment. Mean separation was done using the least significant difference test (LSD) at α = 0.05.

## 3 Results

### 3.1 Laboratory study

Both nutrient priming treatment and soaking duration had significant effects (P< 0.05) on all seed germination indices. Significant interaction effects (P< 0.05) were observed only on GR and MGT.

#### 3.1.1 Germination energy

**Figure 1:**
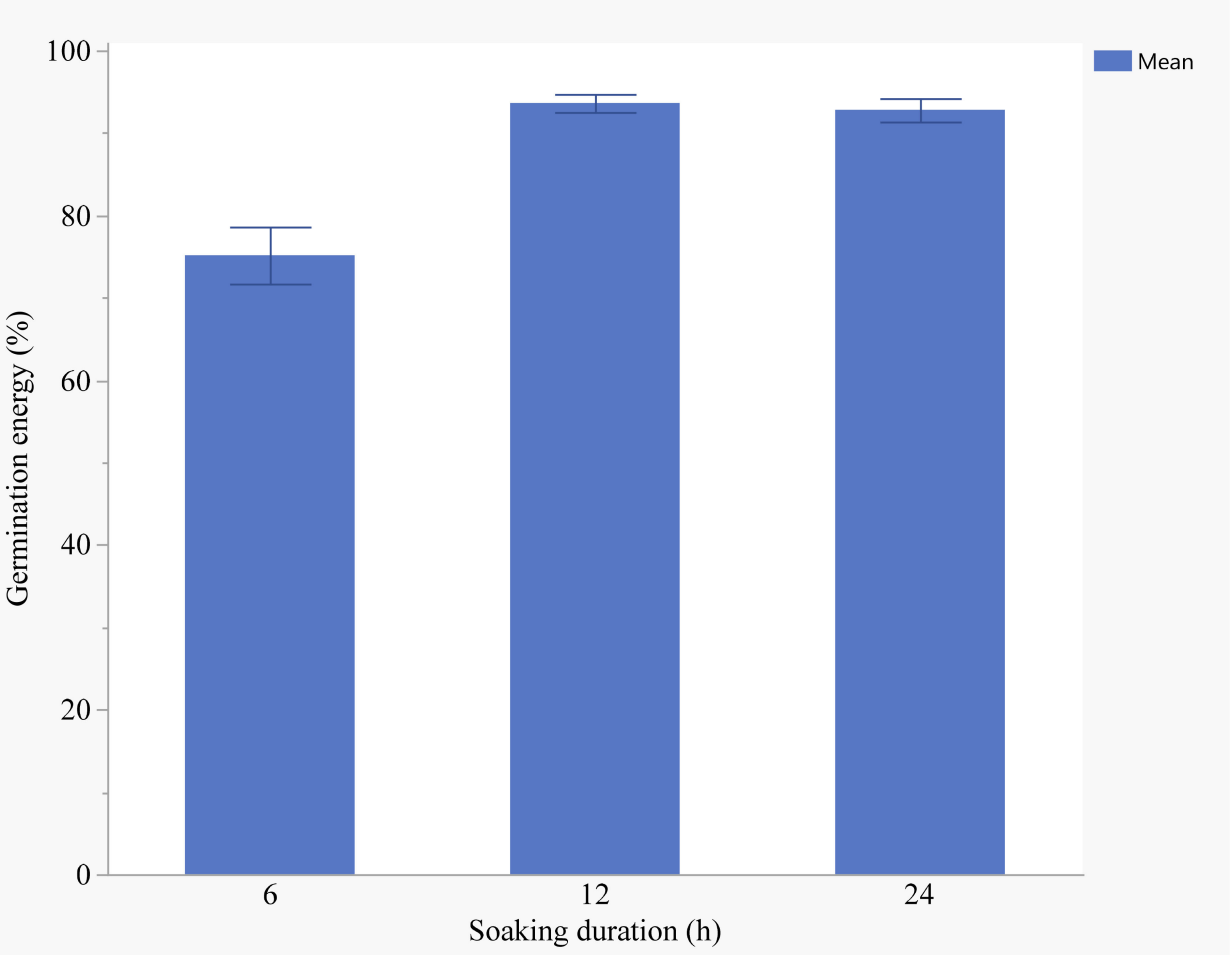
Effects of soaking duration on germination energy (Error bars represent standard error).

Both nutrient seed priming and duration had significant effects on germination energy (GE) (**Table 1**). A significant effect of priming treatments on GE was observed (p< 0.05), Germination energy was significantly higher in seeds treated with 10 mg/L ZnO compared to 20 mg/L ZnO, showing a 62.23% increase (**Table 2**). Similarly, GE was significantly higher in seeds soaked in 10 g/L KNO_3_ compared to 2.5 g/L KNO_3_, 10 mg/L ZnO and 20 mg/L ZnO with mean values of 48.9%, 36.29%, 34.44% and 29.63%, respectively (**Table 2**). Priming duration had a significant (p< 0.05) effect on GE with seeds primed for 24 hours exhibiting the highest GE (45.5%) compared to those primed of 6 hours (30.83%) and 12 hours (39.58%) (**Figure 1**).

**Table 1:** ANOVA table of the effect of nutrient concentration, duration and interaction on Germination Rate (GR), Germination Energy (GE), Mean Germination Time (MGT) and Germination Rate Index (GRI).

Treatment	DF	GR	GE	MGT	GRI
Duration	2	<,0001**	0,0098**	0,0013**	0,0175*
Nutrient	7	<,0001**	0,0043**	<,0001**	<,0001**
Nutrient × duration	14	0,0007**	0,1798	0,0024**	0,0611

Asterisks indicate significant effect at **P < 0.001, *P < 0.05.

**Table 2:** Effect of Nutrient concentration and duration on germination energy.

Nutrient Concertation	Germination energy (%)
ZNO 20 mg/L	29,63^c^
ZNO 10 mg/L	34,44^bc^
ZNO 15 mg/L	47,70^ab^
ZNO 5 mg/L	38,89^abc^
KNO_3_ 10g/L	48,9^a^
KNO_3_ 5 g/L	41,1^ab^
KNO_3_ 2.5 g/L H_2_O (control)	36,29^bc^ 28,14^c^
Duration	Germination energy (%)
24 h	42,5^a^
12 h	39,59^a^
6 h	30,83^b^

Mean with different superscripts indicate significant differences at P < 0.05.

#### 3.1.2 Germination rate

**Figure 2:**
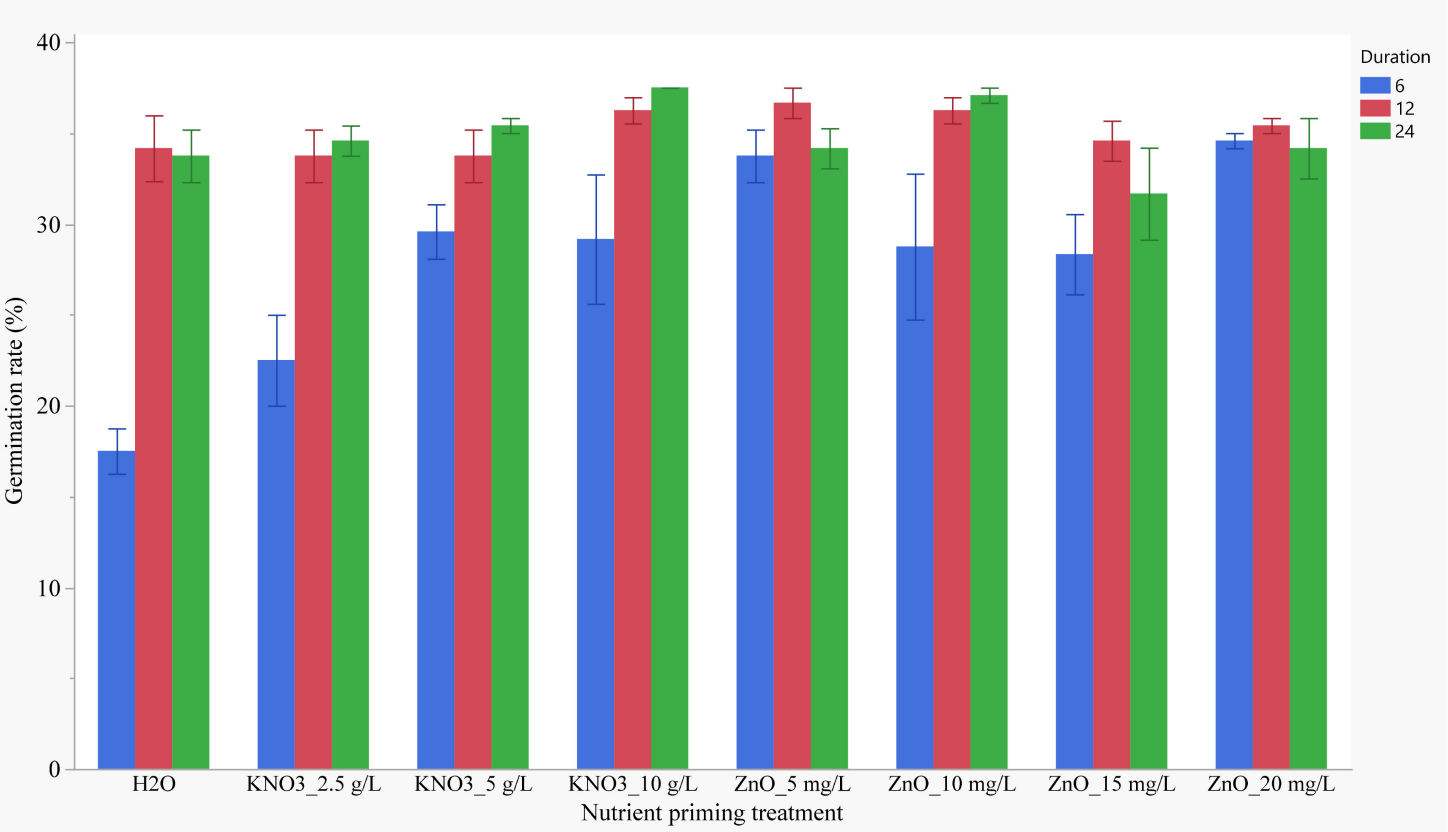
Effects of nutrient seed priming treatment and soaking duration on germination rate (Error bars indicate standard error).

Seed priming with KNO_3_ 10 g/L significantly increased germination rates by 29.96% compared to the control. The optimal priming duration varied depending on the nutrient type (**Figure 2**). For KNO_3_, soaking seeds for 24-hours resulted in the highest germination rate, while soaking for 12-hour was most effective for ZnO. There was significant interaction between priming duration and nutrient seed priming (NSP) concentration on Germination rate (GR) (P<0.001). The lowest GR was observed for control treatment with a 6-hour soaking duration, followed by 2.5 mg/L KNO_3_ for the same duration. The highest GR for the 6-hour soaking duration was observed after priming with 20 mg/L ZnO, but this was not significantly different from 5 mg/L ZnO. For the 12- and 24-hour priming durations, germination rates were similar across most treatments, including the control.

#### 3.1.3 Mean germination time

**Figure 3:**
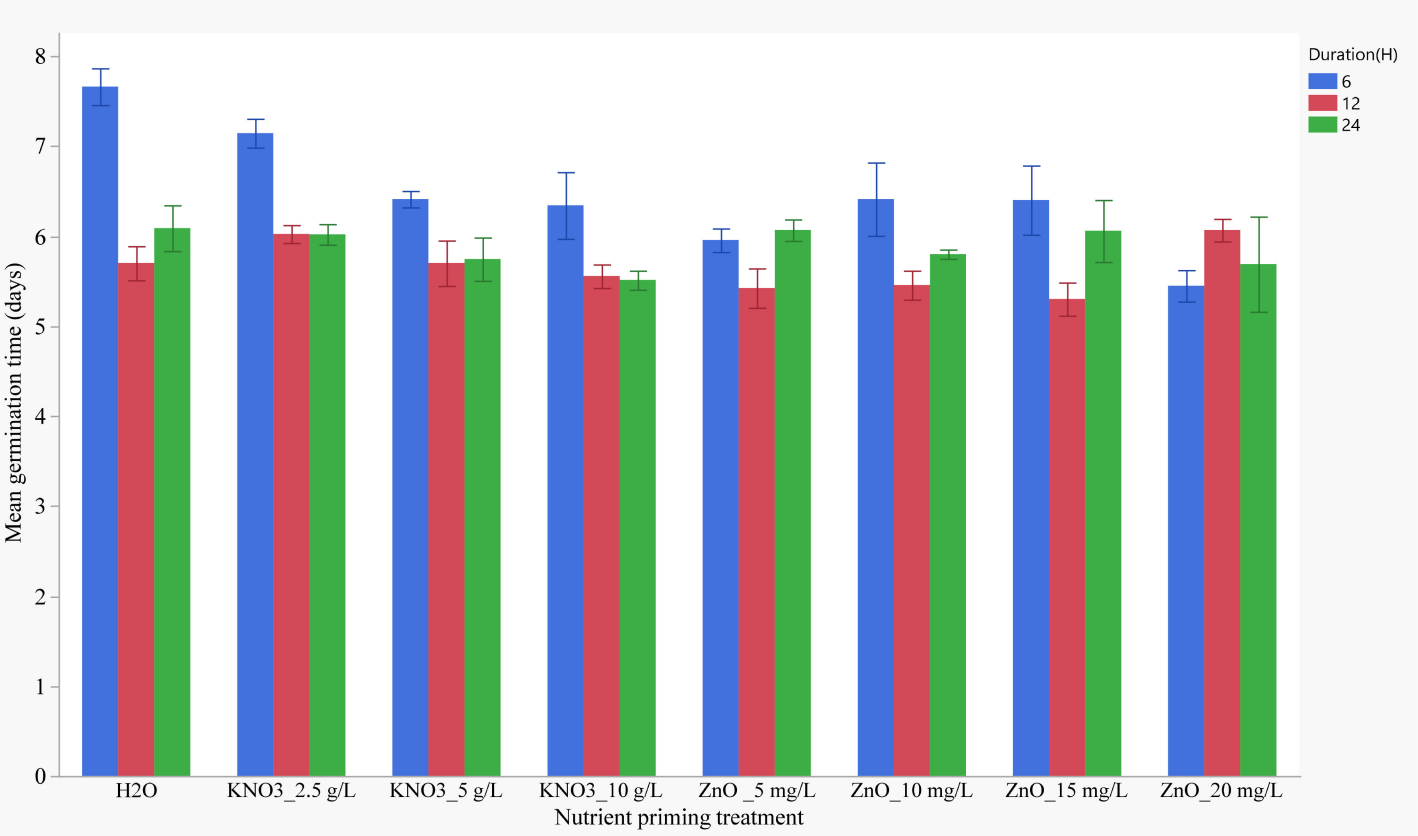
Effects of nutrient seed priming treatment and soaking duration on mean germination time (Error bars indicate standard error).

Mean germination time ranged from 5.3 to 7.6 days across all treatments. For the 6-hour duration, priming with water (control) resulted in the longest MGT, while priming with 20 mg/L ZnO resulted in the shortest MGT. Generally, soaking seeds for 6 hours resulted in longer MGTs than soaking for 12 and 24 hours. The longest germination time was recorded for the control (H_2_O for 6 hours), while the shortest MGT was observed in seeds primed with 15 mg/L ZnO for 12 hours (**Figure 3**).

#### 3.1.4 Germination rate index

**Figure 4:**
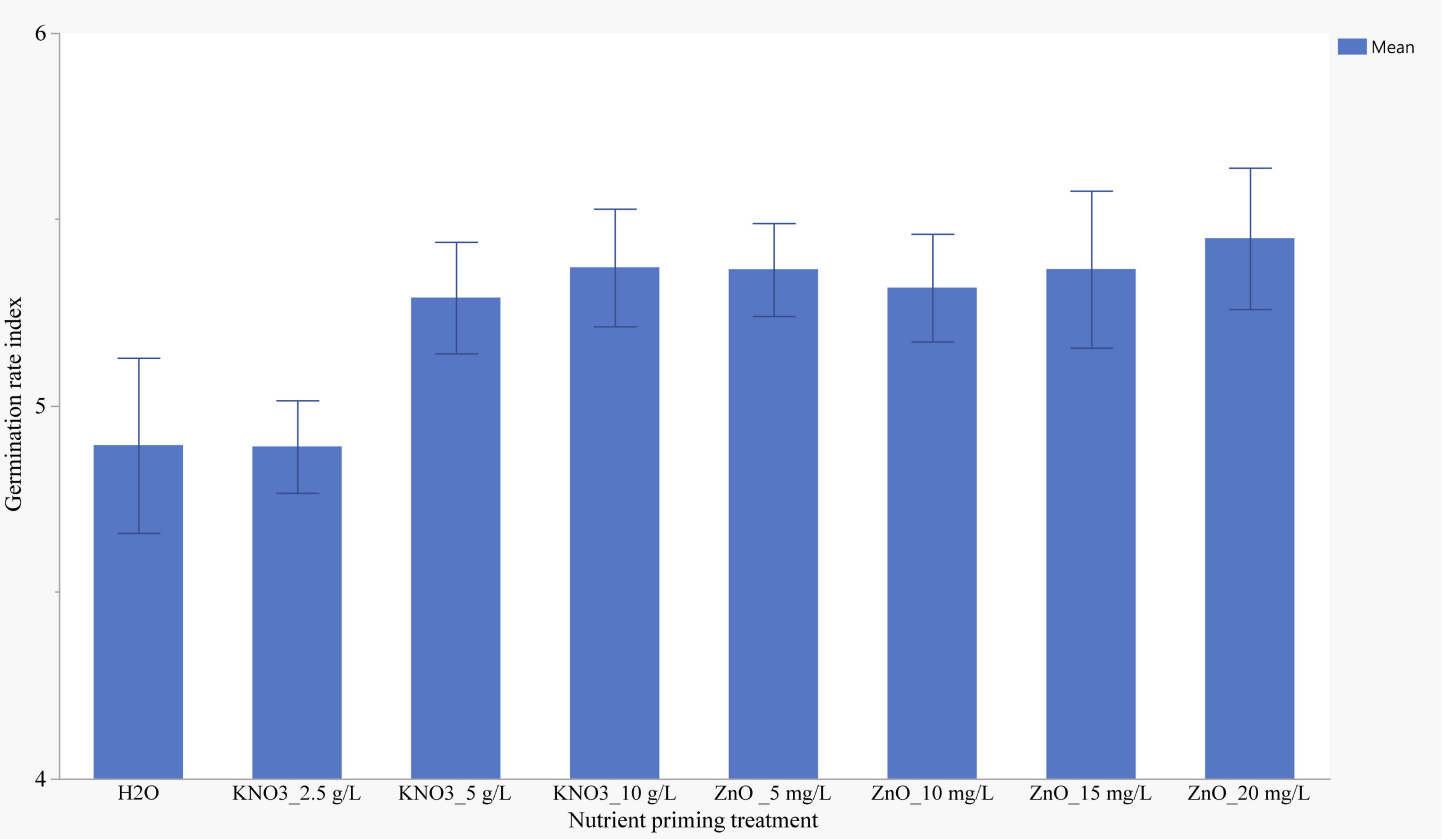
Effects of nutrient seed priming on germination rate index (Error bars indicate standard error).

**Figure 5:**
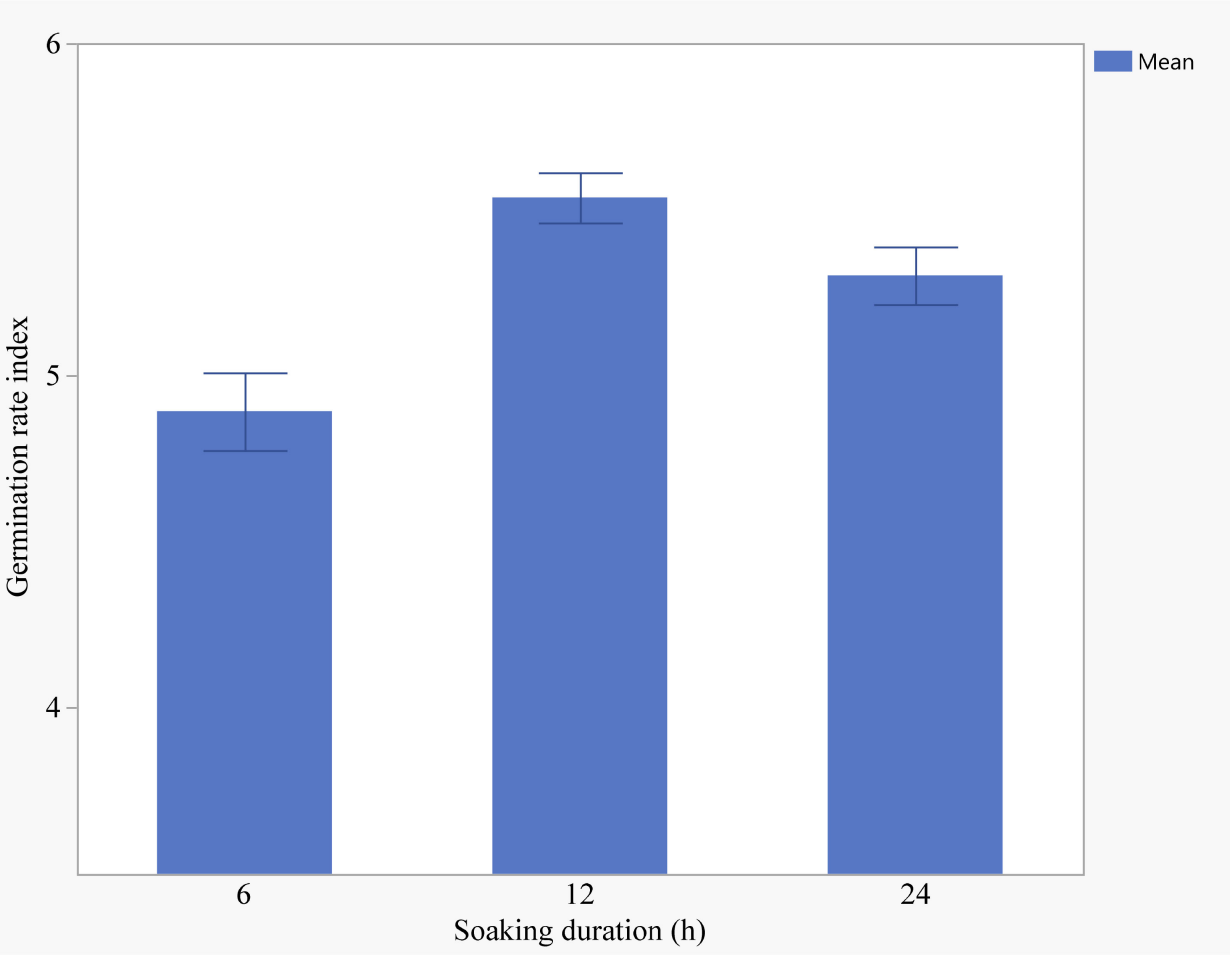
Effects of soaking duration on germination rate index (Error bars indicate standard error).

Seeds primed with 2.5 g/L KNO_3_ and the control group had the lowest germination rate index of 4.89 (**Figure 4**). Seeds primed with 20 mg/L ZnO exhibited the highest GRI, although the difference was not statistically significant compared to other treatments. Soaking seeds for 12 hours resulted in the highest GRI (5.53), followed by soaking for 24 hours (5.30), with the lowest GRI observed after 6 hours of soaking (4.89) (**Figure 5**).

### 3.2 Glasshouse study

The initial chemical properties of the experimental soil are presented in **Table 3**. The soil had a neutral pH of 7.25 and the concentrations of zinc (Zn) and potassium (K) were 4.03 mg/kg and 140 mg/kg, respectively.

**Table 3:** Some chemical characteristics of the soil used.

Property	Value
Total C (%)	0.47
Total N (%)	0.038
Available P (mg/kg)	11.95
Available Na (mg/kg)	70.6
Exchangeable Na (cmol/kg)	0.3071
Available K (mg/kg)	140
Exchangeable K (cmol/kg)	0.3581
Available Ca (mg/kg)	1640
Exchangeable Ca (cmol/kg)	8.1836
Available Mg (mg/kg)	711
Exchangeable Mg (cmol/kg)	5.8519
RESIST (Ohms)	700
pH	7.25
Zn (mg/kg)	4.03
Clay (%)	28
Sand (%)	64
Silt (%)	8

#### 3.2.1 Influence of nutrient seed priming concentration and duration of on seedling emergence indices under glasshouse conditions

Nutrient seed priming and priming duration significantly affected seedling emergence indicated by germination energy (GE) (**Table 4**) The analysis revealed a significant effect of priming duration on GE (p< 0.001). Seeds primed for 24-hours exhibited the highest GE (85.6%) compared to those primed for 6 hours (78.2%) and 12 hours (80.9%) (**Figure 1**). A clear trend of increasing seedling emergence rates was observed with longer priming durations, with the 24-hour soaking treatment showing the most significant improvement. Seedling emergence rates increased with longer priming durations, with the 24-hour soaking treatment showing the highest emergence rate (p< 0.001) (**Figure 1**)

**Table 4:** Effects on germination and seedling emergence indices.

Treatment	DF	GR (%)	GE (%)	DE (days)	EE	MET
Nutrients	4	0,2196	0,0021*	0,1064	0,0844	0,1075
Duration	2	0,0594	0,0001*	0,3189	0,3123	0,5083
Nutrient*Duration	8	0,5502	0,1001	0,5481	0,7318	0,8585

*Indicates significant difference at P < 0.05.

#### 3.2.2 Influence of nutrient seed priming concentration and duration of chillies on seedling growth parameters

Nutrient seed priming significantly influenced (p< 0.001) multiple early growth parameters of chilli seedlings, under glasshouse conditions including shoot height (SH), root length (RL), fresh seedling weight (FSW) and steam diameter (SD), while priming duration only had a significant effect on FSW (**Table 5**). There was no significant interaction between these two factors on any of the measured parameters.

**Table 5:** ANOVA table presents the effect of nutrient seed priming duration, nutrient type and their interaction on the measured parameters.

Treatment	DF	NO: leaves	SH (cm)	RL (cm)	FSW (g)	DSW (g)	SD (mm)	CCI (SPAD)
Nutrients	4	0,2098	0,0036*	0,0216*	0,0266*	0,1646	0,0027*	0,4592
Duration	2	0,1045	0,0839	0,7901	0,0165*	0,1758	0,0731	0,8937
Nutrient*Duration	8	0,0988	0,2486	0,1400	0,0869	0,0862	0,6078	0,5255

*Indicates significant difference at P < 0.05. Where: GR, germination rate; GE,germination energy; SH, shoot height; RL, root length; FSW, fresh shoot weight; DSW, dry shoot weight; SD, stem diameter; CCI, chlorophyll content index; DE, days to emergence; EE,; MET, mean emergence tim.

##### 3.2.2.1 Shoot length

**Figure 6:**
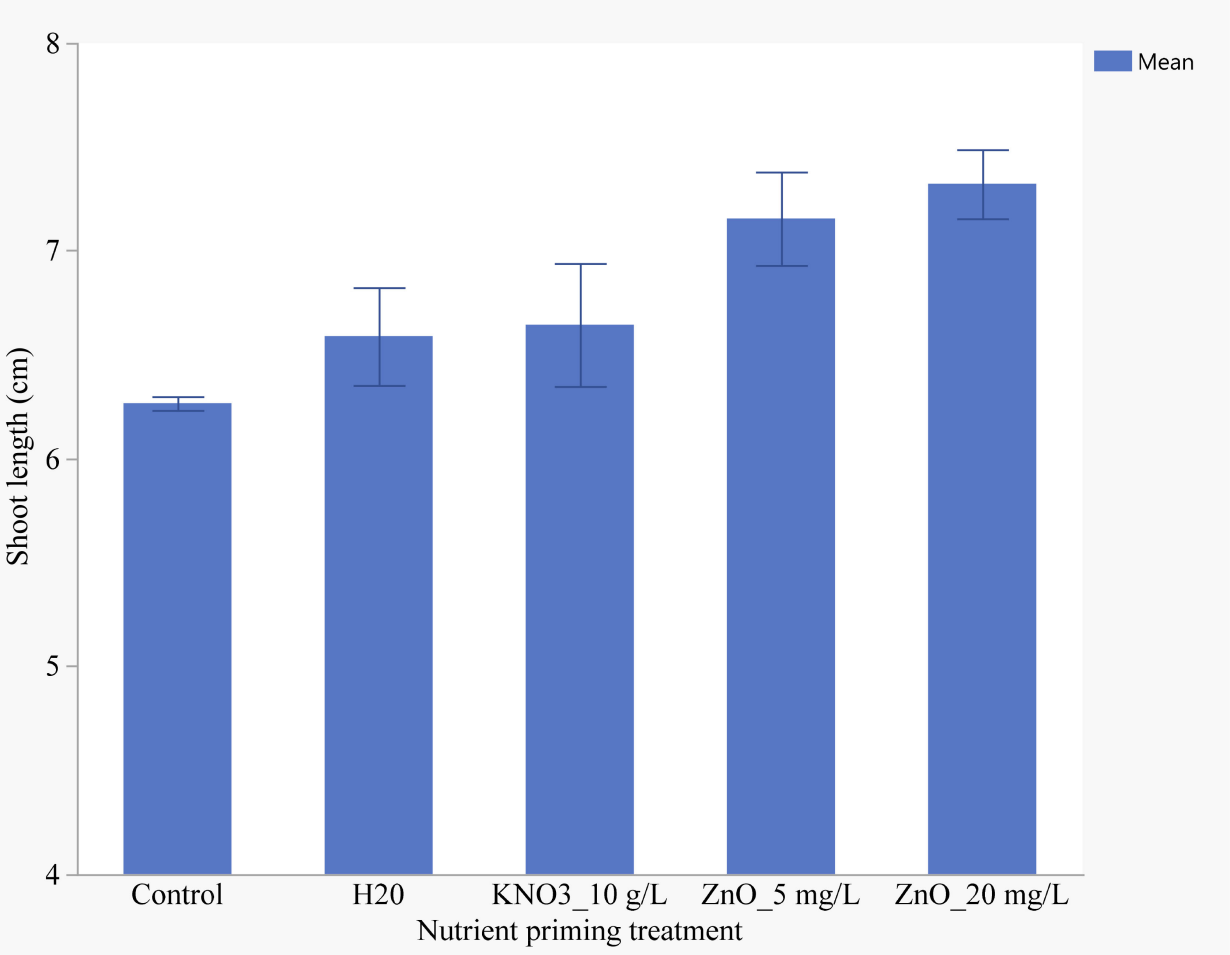
Effects of nutrient seed priming on shoot length (cm) (Error bars indicate standard error).

Seedlings primed with 20 mg/L ZnO exhibited the highest shoot length (7.32 cm) compared to other concentrations (**Figure 6**). However, no significant differences were observed in shoot length between seedlings primed with 10 mg/L and 20 mg/L Zn. Unprimed seedlings had the shortest shoot length, but this was not significantly different from those primed with water or KNO.

##### 3.2.2.2 Root length

**Figure 7:**
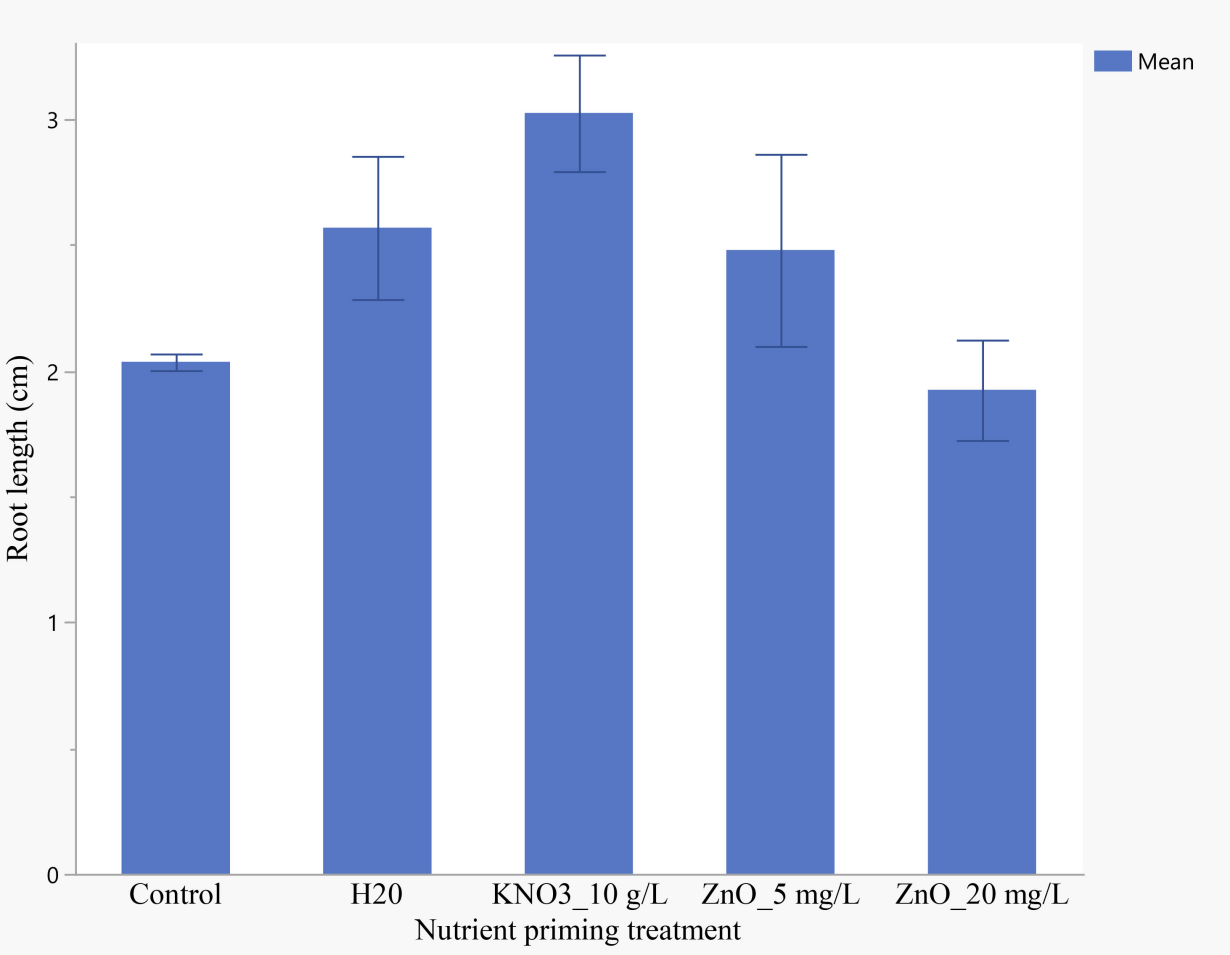
Effects of nutrient seed priming on root length (cm) (Error bars indicate standard error).

Seedling primed with 10 g/L KNO_3_ developed significantly longer roots than those primed with 20 mg/L ZnO. However, no significant differences were observed among seedlings primed with H_2_O, 10 g/L KNO_3_, 5 mg/L ZnO and unprimed control (**Figure 7**). Seed primed with 10 g/L KNO_3_ produced seedling with an average of 3.02 cm long roots and ZnO 20 mg/L seedlings had the shortest roots averaging 1.9 cm.

##### 3.2.2.3 Fresh seedling weight

**Figure 8:**
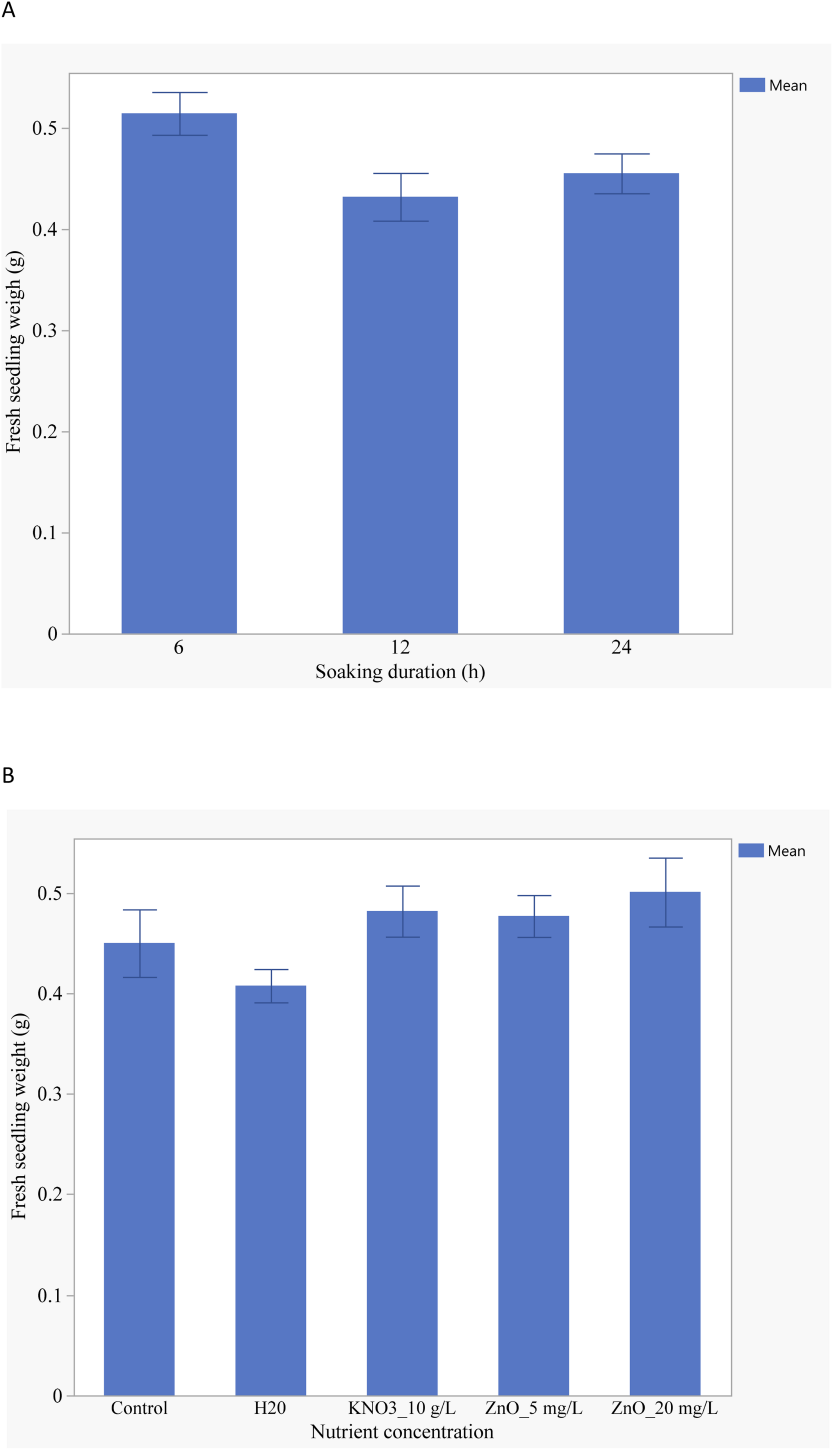
**(A)** Effects of soaking duration on fresh seedling weight; **(B)** Effects of nutrient seed priming on fresh seedling weight.

Both nutrient seed priming and priming duration significantly influenced fresh seedling weight. Seeds primed for 6 hours exhibited the highest fresh weight (0.5 g), followed by those primed for 24 hours (0.4 g) (**Figure 8A**). A ZnO concentration of 20 g/L significantly impacted fresh seedling weight (**Figure 8B**) demonstrating the positive influence of zinc on early growth. No interaction was observed between nutrient seed priming treatments and soaking duration on fresh seedling weight suggesting that the effects of these factors on seedling growth are largely independent.

##### 3.2.2.4 Stem diameter

**Figure 9:**
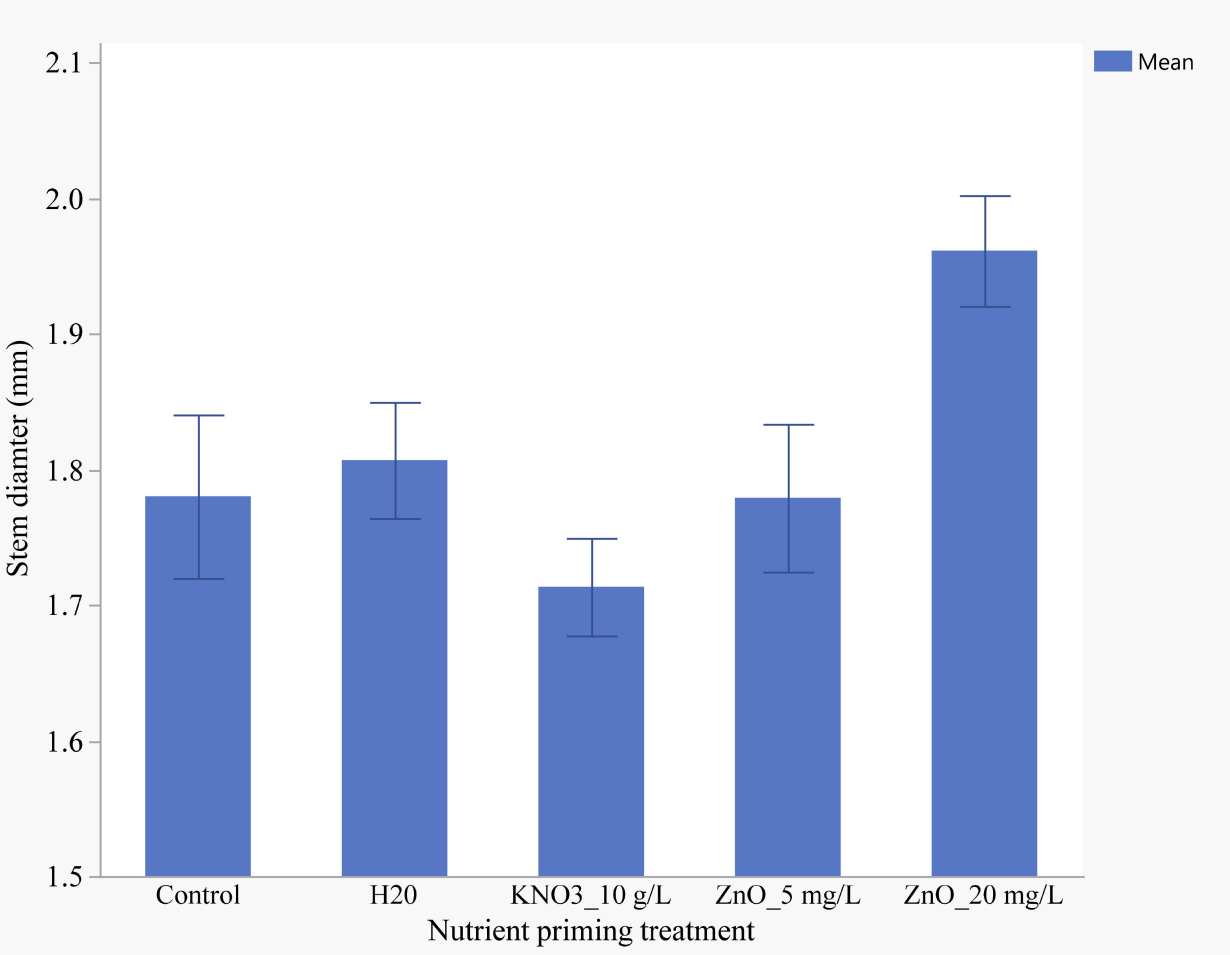
Effects of nutrient seed priming on stem diameter (cm) (Error bars indicate standard error).

Nutrient seed priming had a significant effect on stem diameter (p< 0.05). The thickest stems (1.96 mm) were observed in seedlings primed with 20 mg/L ZnO, while the thinnest stems (1.71 mm) were found in those primed with KNO_3_ (**Figure 9**).

## 4 Discussion

Priming with ZnO at 15 mg/L and KNO_3_ at 10 mg/L resulted in the highest germination energy levels, while lower levels were observed for other concentrations. These findings highlight the positive effect of ZnO on germination and the importance of optimal nutrient concentrations for seed germination and early seedling growth. Studies have shown that specific nutrients, such as KNO_3_, can promote germination in various plant species by providing essential nutrients for metabolic processes (Rizk et al., 2022; Miladinov et al., 2020; Vinolina and Stefanie, 2024). In addition to providing essential nutrients, priming can enhance enzymatic activities involved in seed germination, leading to higher germination energy levels (Ali et al., 2011). Bhat et al. (2023) and Santika et al. (2022) suggested that nutrient concentrations impact seed coat permeability and dormancy contributing to the observed results. Soaking seeds in KNO_3_ solutions has been shown to soften seed coats, promoting quicker germination processes, The enhanced permeability facilitates better water uptake and nutrient absorption, resulting in improved germination energy levels (Açikgöz and Kara, 2019; Haque, 2024).

The duration of soaking plays an important role in seed germination outcomes. Longer exposure times allow seeds to absorb essential nutrients, leading to higher germination energy levels (Javed et al., 2020; Eremrena and Mensah, 2016). However, shorter durations or excessive nutrient concentrations can inhibit seed germination (Ribeiro et al., 2019). Seeds primed for 24 hours exhibited the highest GE suggesting that extended priming enhances imbibition and metabolic activation, resulting in a higher proportion of rapidly and uniformly germinating seeds. These findings align with Ruttangavungboworn et al. Who identified 24-hours as the optimal priming duration for maximizing GE regardless of the nutrient type, this indicates that a longer priming duration may be necessary to fully prime the seeds and unlock their germination potential.

Similar to GE, both nutrient type and priming duration significantly affected GR. Priming with KNO_3_, at a concentration of 10 mg/L for 24 hours resulted in significantly higher GR compared to the control and other treatments. This finding is consistent with previous research demonstrating the effectiveness of KNO_3 in_ enhancing the germination rates of chillies. The improved GR observed with priming with KNO_3_ can be attributed to its role in activating enzymes involved in the early stages of germination and enhancing water uptake and imbibition (Farooq et al., 2020). The interaction between nutrient type and priming duration further highlights the importance of optimizing both factors to maximize GR. The positive effect of KNO_3_ on GR is due to its provision of readily available potassium ions, which are essential for cell elongation and division during germination. In contrast, the lower GR observed with ZnO could be due to the potential toxicity of high Zn concentrations, which can inhibit germination-related enzyme activity and impair water uptake. The relationship between nutrient concentration and priming duration in seed germination processes highlights the complexity of the mechanisms involved and the need for tailored nutrient management strategies to optimize germination outcomes. The significant interaction effect between nutrient concentration and priming duration on GR shows the importance of optimizing both factors. While a 24-hour priming duration was optimal for KNO_3_ at 10 g/L, a shorter duration of 12 hours proved more effective for ZnO 10mg/l. This difference could be related to the specific mechanisms by which these nutrients influence germination processes.

Priming with 20 mg/L ZnO reduced MGT compared to the control and KNO_3_ treatments. This finding suggests that ZnO priming can accelerate the germination process, leading to faster and more uniform seedling emergence. The shorter MGT observed with ZnO priming could be attributed to its role in enhancing metabolic activity and promoting the mobilization of reserves required for germination. Consistent with trends observed for other germination indices, a 12-hour priming duration resulted in the shortest MGT for both KNO_3_ and ZnO. This indicates that an optimal priming duration, neither too short nor too long is crucial for maximizing metabolic activation and physiological preparation for germination Shahein and El-Gaafarey (2021). Additionally, nutrient seed priming, with 20mg/L ZnO significantly enhanced the germination index of chili seeds compared to the control. The germination index, which considers both the rate and completeness of germination, was highest in seeds primed with ZnO 20mg/l for 12 hours.

The present results demonstrate the potential of nutrient seed priming to improve the germination performance of chili peppers. Priming with KNO_3_ and ZnO enhanced various germination indices at different levels, depending on the nutrient and duration used (Patel and Patel, 2020). ZnO generally outperformed KNO_3_ in terms of germination rate, mean germination time, and germination index. This can be attributed to the unique roles of these nutrients in seed physiology and metabolism. Potassium is an essential macronutrient involved in enzyme activation, osmoregulation, and protein synthesis, all crucial for seed germination (Patel et al., 2020). Zinc micronutrient also plays an important role in membrane integrity, protein synthesis, and antioxidant defense, which can also contribute to enhanced germination (Rai-Kalal and Jajoo, 2021).

The observed interaction between nutrient types and priming duration suggest that the optimal combination may vary depending on the crop and the targeted germination parameters. Determining the most suitable priming treatment can help improve the establishment and productivity of chili pepper crops (Yafizham and Herwibawa, 2018). Research has demonstrated that primed seeds can retain stress memory, allowing them to activate cellular defense mechanisms earlier, reduce imbibition time, increase germination promoters, and regulate osmotic balance, thereby influencing germination outcomes. The pathways involved in reserves mobilization during germination may vary among various crops, underscoring the diverse responses to nutrient concentrations.

Nutrient seed priming significantly improved several growth parameters of chilli seedlings compared to the control as shown by the data presented in **Table 4** and supported by various previous studies (Patel et al., 2020). The observed improvements can be attributed to the availability of ZnO during the critical early stages of crop development, which stimulates various physiological and biochemical processes. Priming with 20 mg/L ZnO produced the highest shoot length (7.32 cm) and fresh biomass (4.2 g), highlighting the importance of zinc as a micronutrient essential for early seedling development. Zn plays an important role in enzyme activation, protein synthesis, and chlorophyll formation. The increased availability of Zn in ZnO-primed seedlings likely contributed to the enhanced growth observed in this study (Patel et al., 2020). Nutrient priming, particularly with ZnO 5mg/l, significantly improved seedling vigor index, an essential measure of seedling growth and early development. These findings demonstrate that nutrient priming can enhance early growth, photosynthetic efficiency, and nutrient uptake in seedlings. The improved seedling vigor observed in the primed seedlings emphasizes that nutrient priming can result in a distinct advantage in the early stages of plant growth, potentially leading to enhanced establishment and performance in the field (Patel et al., 2020). No statistically significant effects were noted on the germination rate (GR) due to treatment duration and nutrient. This suggests that while nutrient seed priming improves post-germination growth, it does not necessarily speed up or improve the initial germination process.

Priming duration also influenced seedling growth, with varying durations resulting in distinct impacts. Specifically, extended priming durations promoted increased fresh shoot weight (FSW) indicating enhanced vegetative growth and biomass accumulation consistent with Stephan et al. (2020). This suggests that prolonged exposure to the priming solution, allows for continuous absorption of nutrients. Similar to observations in maize and millet (Patel et al., 2020), our study found that NSP positively influenced chilli seedling growth. Previous research corroborates our findings, demonstrating that longer priming durations coupled with nutrient availability, lead to increased shoot height and biomass. This is likely due to more efficient resource allocation towards cell expansion and division over extended treatment periods resulting in higher fresh biomass accumulation. Nciizah et al. (2020) also noted that prolonged exposure to higher nutrient concentrations improved nutrient absorption, allowing plants to utilize accessible nutrients more effectively for growth.

No significant interaction was observed between priming duration and nutrient type observed. This indicates that their effects are largely independent. Consequently, the optimal priming duration appears consistent across different nutrient types, and the choice of nutrient does not significantly alter the priming duration for effective results.

## 5 Conclusion

This study demonstrates the significant impact of nutrient seed priming with KNO_3_ and ZnO on germination indices of chilies. Notably, an interaction effect between nutrient solution and soaking duration highlights the importance of optimizing these factors for specific germination outcomes. Priming with 20 mg/L ZnO generally resulted in higher germination rates and shorter mean germination times compared to priming with KNO_3_ with optimal priming duration 24 hours for KNO_3_ and 12 hours for ZnO under laboratory conditions. Furthermore, glasshouse experiments revealed that nutrient seed priming effectively enhances seedling emergence and early growth and vigor of chillies. Specifically, priming with 20 mg/L ZnO for 6 hours yielded the best results for shoot length and root length while longer priming durations of up to 24 hours were beneficial for fresh seedling weight. These findings highlight the potential of nutrient seed priming, especially with ZnO, as an effective and sustainable agricultural practice to improve seedling growth and vigor in chilli plants. The optimization of priming duration and nutrient type and or concentration, can assist farmers to significantly enhance crop establishment and potentially increase yields. These results provide valuable insights for small-scale farmers seeking to improve chilli seed germination and early growth through nutrient priming.

## Data Availability

The original contributions presented in the study are included in the article/supplementary material. Further inquiries can be directed to the corresponding author.
